# Prosthodontic Rehabilitation of a Completely Edentulous Patient by Salvaging Ailing Implants Using a Bar Retained Over Denture: A Case Report

**DOI:** 10.7759/cureus.63157

**Published:** 2024-06-25

**Authors:** Muness Akhtarkhavari, Praveen Rajagopal, Godwin Clovis Da Costa, Meena Aras, Vidya Chitre, Sadhvi G Naik

**Affiliations:** 1 Department of Prosthodontics and Crown and Bridge, Goa Dental College and Hospital, Bambolim, IND

**Keywords:** implant osseointegration, low profile hader bar, ailing implants, implant supported overdenture, complete edentulism, implantoplasty

## Abstract

Patients who are edentulous experience challenges with their dentures, especially the mandibular ones. The primary concerns of these patients include reduced chewing efficiency, instability, and loss of retention. With the advancement of implants and prosthetic options, these concerns can be addressed by resorting to implant-supported fixed and removable prostheses. The impetuous use of dental implants to solve these issues leads to inadvertent failures in the treatment undertaken. Improper planning of cases leads to prosthesis breakage and implant failures leaving the patient dissatisfied. One such case of rehabilitation of a completely edentulous over-denture patient with ailing implants is described in this clinical report.

## Introduction

Over the years there has been a dramatic shift in the treatment options and preferences for rehabilitation of completely edentulous patients. Conventional complete dentures were the standard treatment earlier; however, they possess several inadequacies, especially in resorbed mandibular ridges. Implant-retained or supported overdentures resolve most of these shortcomings. These dentures fit on top of the implants and partially or completely take support from the implants and their corresponding attachments.

The placement of implants in the bone reduces the amount of residual ridge resorption experienced by the patient and improves muscular activity owing to better stability of the dentures [1,2]. The biting force of these patients is significantly higher which increases their chewing efficiency [3]. This expands the ability to consume food in different forms with various consistencies that pave the way for a diet with better nutritional value that imparts overall positive health [4]. Since the fit of these dentures is superior, better teeth arrangement, in optimal positions improves the appearance of the patient [5].

There is a higher level of general patient satisfaction in comparison with conventional complete dentures, which in turn contributes to the enhancement of their quality of life [6,7]. For implants to function well, they must be osseointegrated to the underlying bone. If this process is hindered due to any reason, the implant tends to fail. When this condition becomes chronic, with minimal signs and symptoms, it is categorized as an ailing implant. This case report describes a conservative yet effective way to salvage the ailing dental implants with an implant retained overdenture using a Hader bar to rehabilitate the patient.

## Case presentation

**Figure 1:**
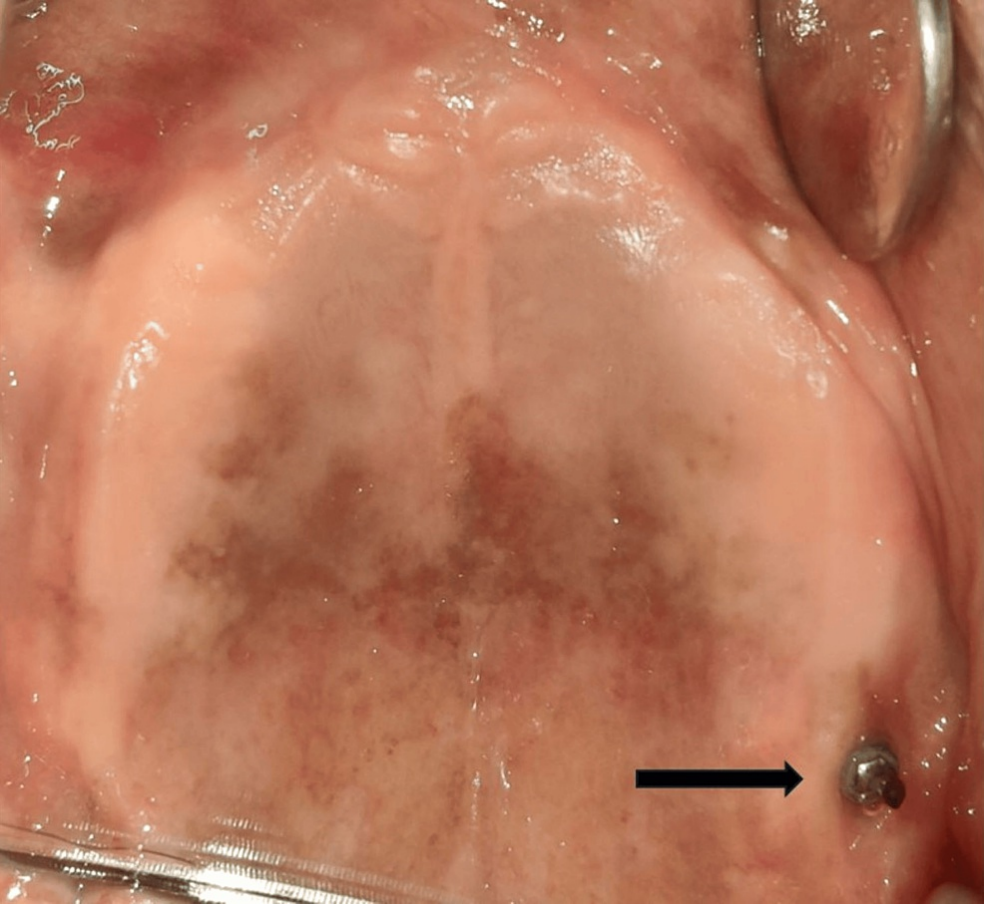
Edentulous maxillary arch with implant in the left maxillary tuberosity area with a stud attachment Arrow showing the single implant with stud attachment

**Figure 2:**
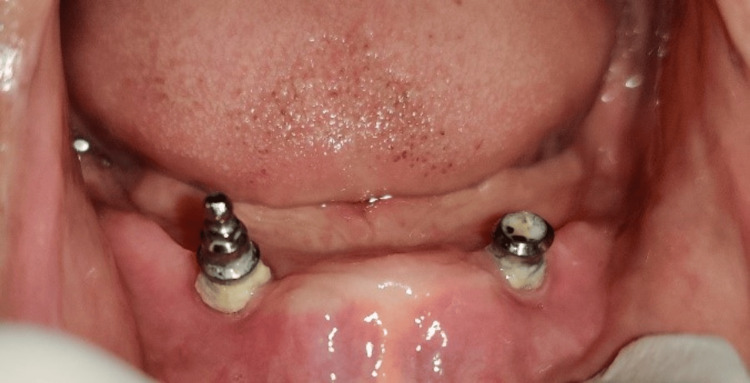
Edentulous mandibular arch with misaligned implants in the bicuspid area with a tenacious band of calculus

**Figure 3:**
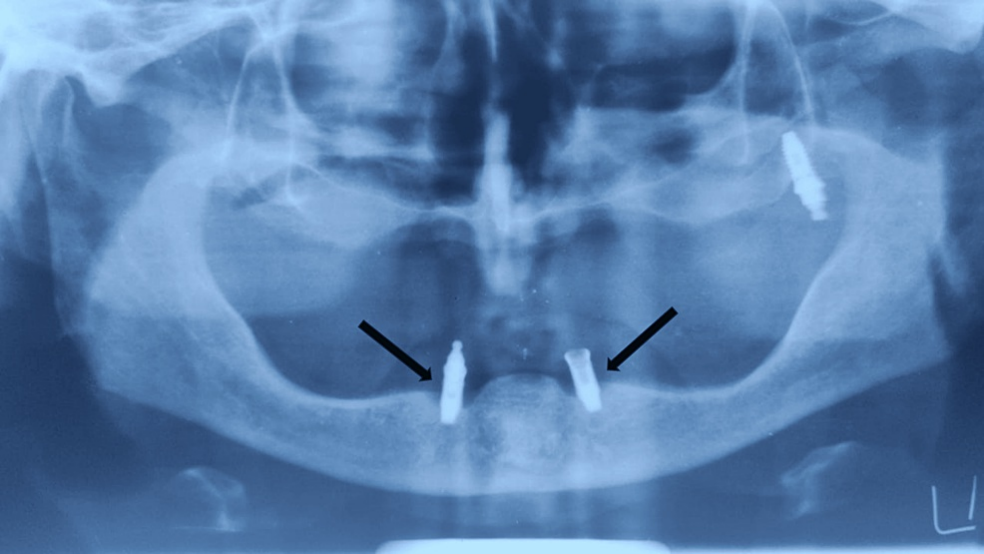
Orthopantomogram showing bone loss up to apical third with respect to the mandibular implants Arrow showing bone loss around left and right mandibular implants

**Figure 4:**
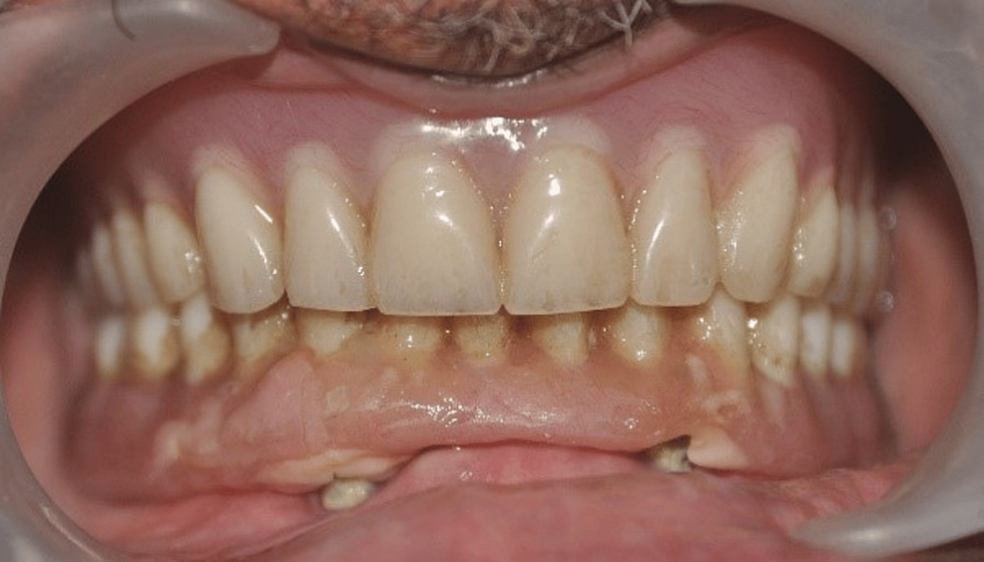
Maxillary and mandibular old dentures with food stains

**Figure 5:**
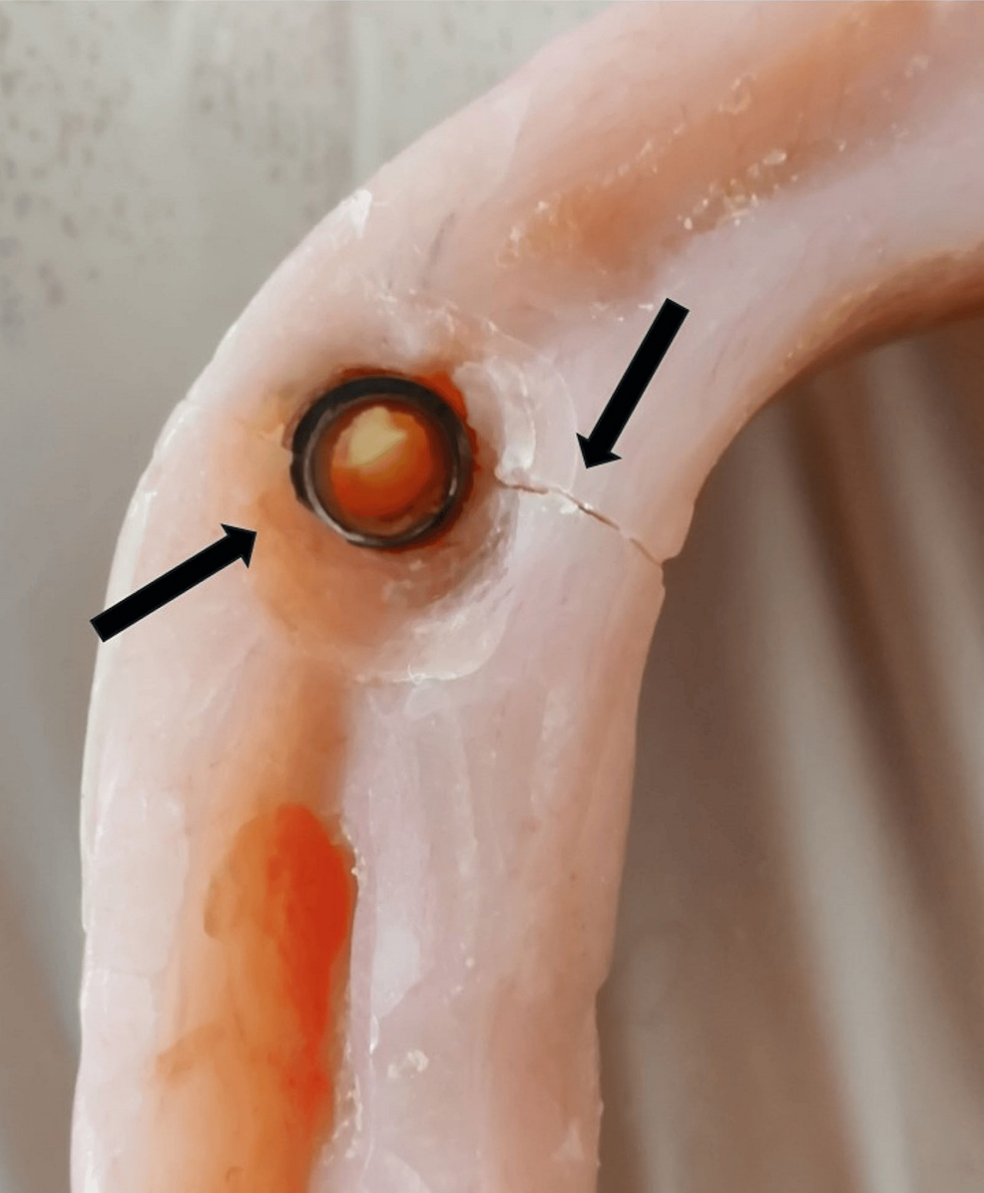
Mandibular old denture with cracks and multiple repair attempts Arrows showing cracks and repair attempts on the mandibular old denture

A 75-year-old patient came to the Department of Prosthodontics and Crown and Bridge, with a chief complaint of ill-fitting dentures. On examination, completely edentulous maxillary and mandibular arches were seen. The patient gave a history of four implant placements eight years ago in the maxillary arch, out of which three implants failed and fell out. In the maxillary arch, there was a single implant noted in the left maxillary tuberosity area with a stud abutment which was in good condition (Figure 1). In the mandibular arch, two similar misaligned implants were seen in the bicuspid region. The stud abutment of the mandibular left implant had loosened and was misplaced. A thick, tenacious band of calculus was seen extending up to the gingival margin (Figure 2). An orthopantomogram showed severe bone loss till the apical third of the mandibular implants (Figure 3). The implants were not mobile and did not show any signs of peri-implantitis. The patient’s old dentures presented with food stains, evidence of cracks, and multiple repair attempts (Figures 4, 5).

**Figure 6:**
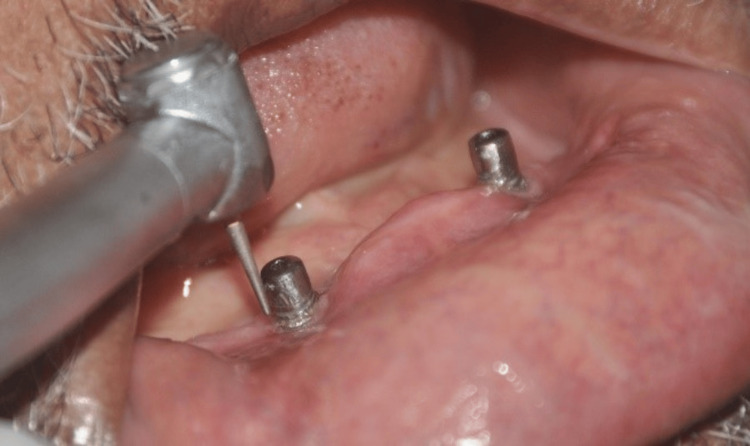
Implantoplasty of the mandibular implants done using a fine diamond polishing bur

**Figure 7:**
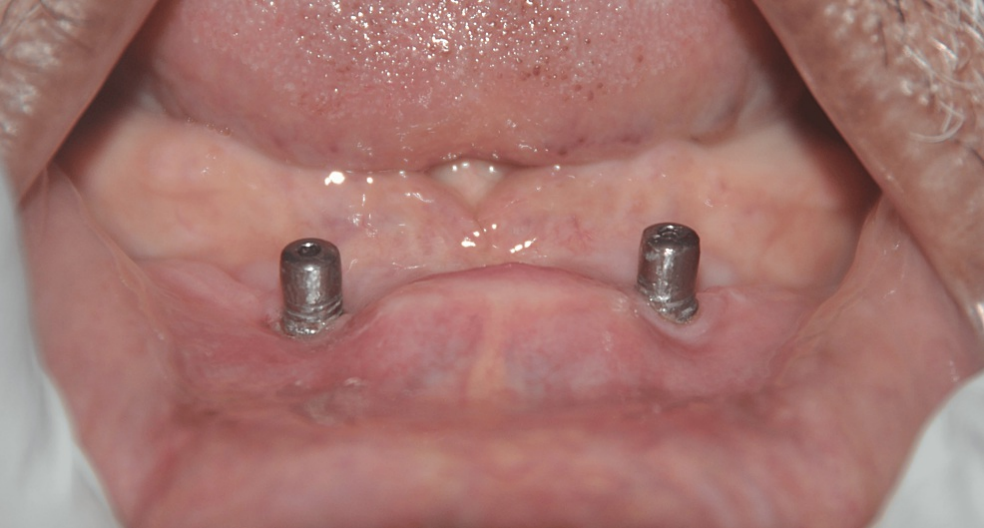
Correction of misalignment of implants

A meticulous scaling and implant surface debridement was done using plastic implant curettes (Hu-Friedy). A tentative jaw relation was done after which it was decided that the height of the mandibular implants was excessive. The stud abutment of the mandibular right implant was removed, and cover screws were placed on both the implants. Implantoplasty of both mandibular implants using a fine diamond polishing bur was performed to smoothen out the threads and correct the malalignment (Figures 6, 7).

**Figure 8:**
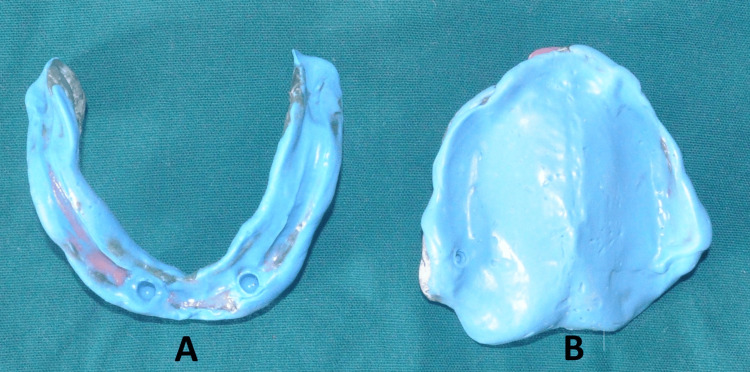
Mandibular and maxillary final impressions. A: mandibular, B: maxillary

Measurement of the vertical dimension was done on the articulated dentures to assess the space for attachments. Primary impressions of the maxillary arch were made with impression compound (MAARC Dental Impression compound, Shiva Products, Mumbai, India) and dental alginate (Tropicalgin, Zhermack, Germany) was used for the mandibular arch. The final impression of both the maxillary and mandibular arches was made with green stick (Samit Tracing Sticks, Dento Kem, Ballabgarh, India) and Polyvinyl siloxane light body (Zhermack, Germany) material (Figure 8).

**Figure 9:**
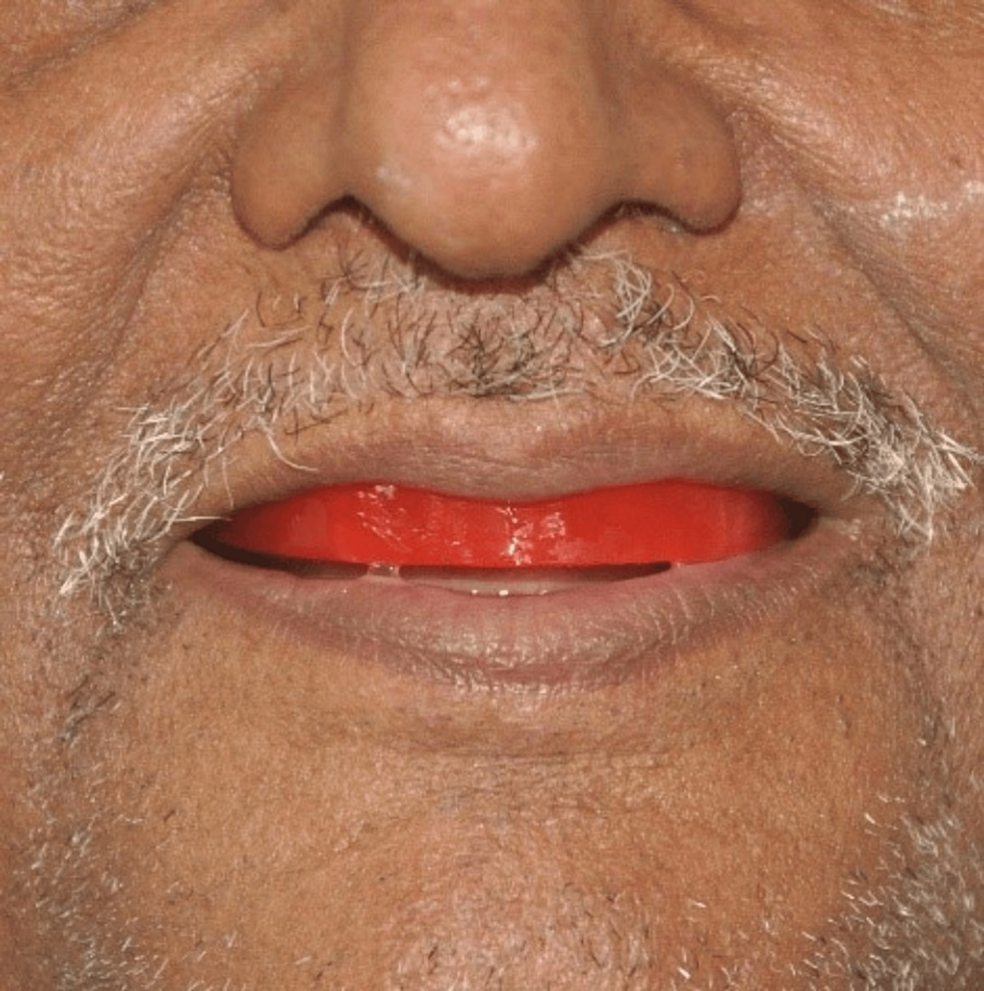
Jaw relation

**Figure 10:**
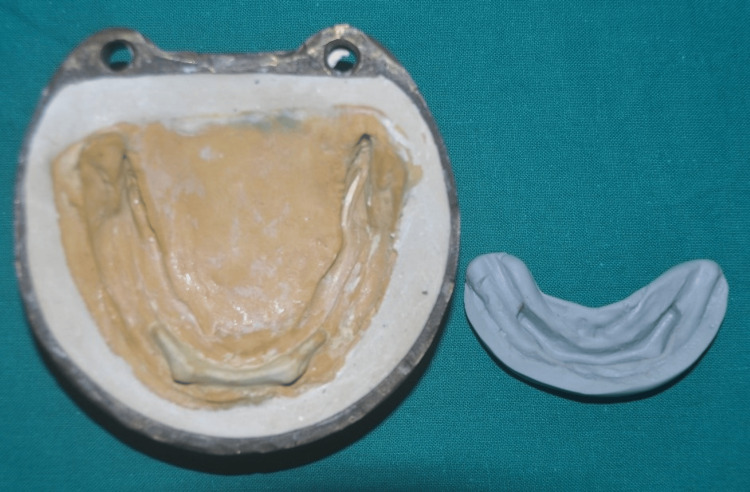
Putty index of the Hader bar

**Figure 11:**
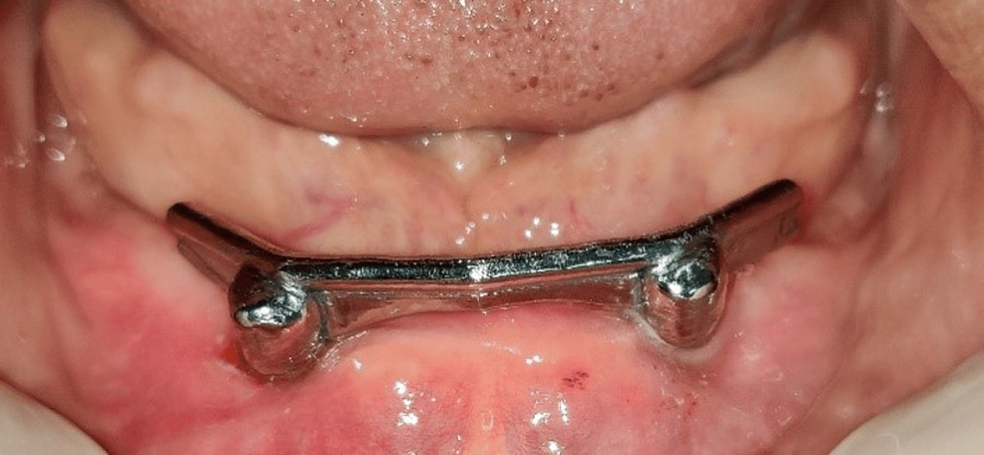
Cementation of the Hader bar

**Figure 12:**
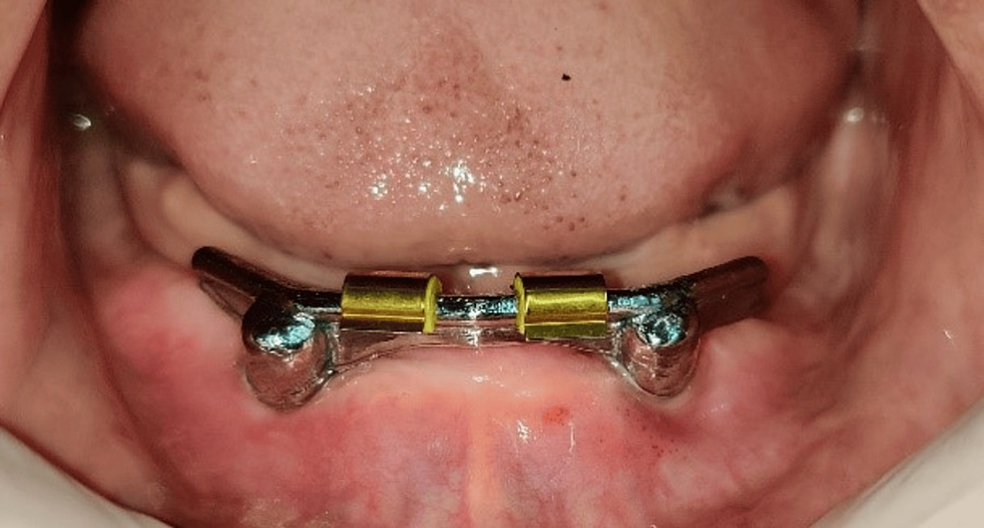
Placement of clips on the Hader bar

**Figure 13:**
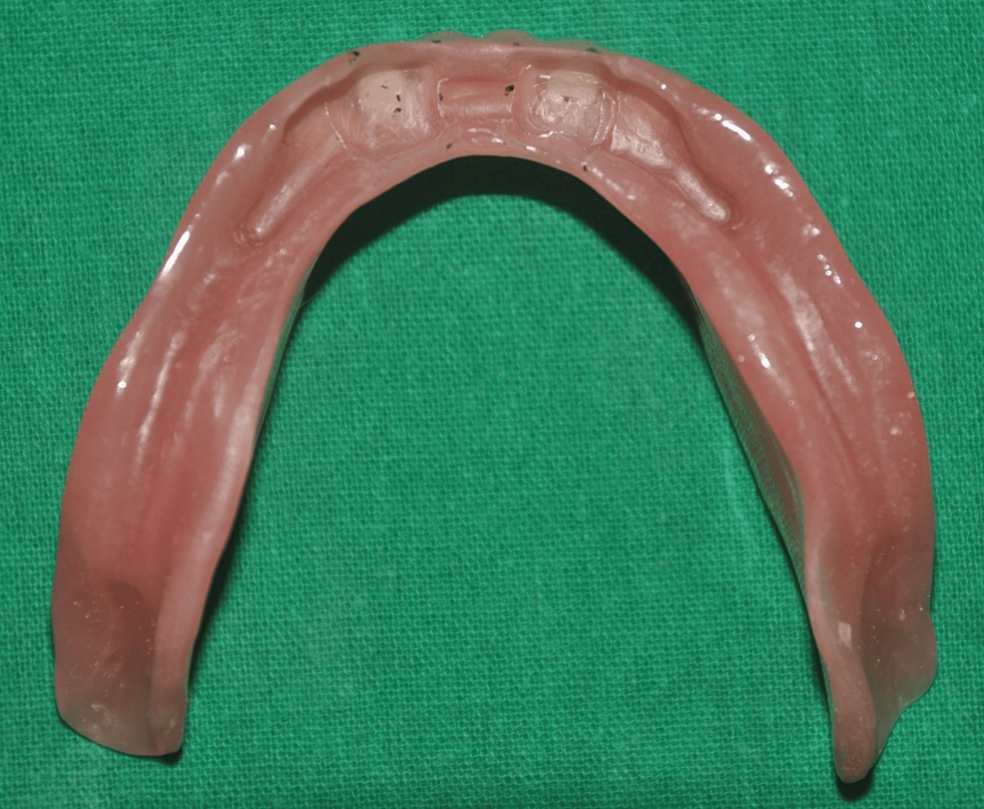
Space created in the lower denture to accommodate the Hader bar clips

**Figure 14:**
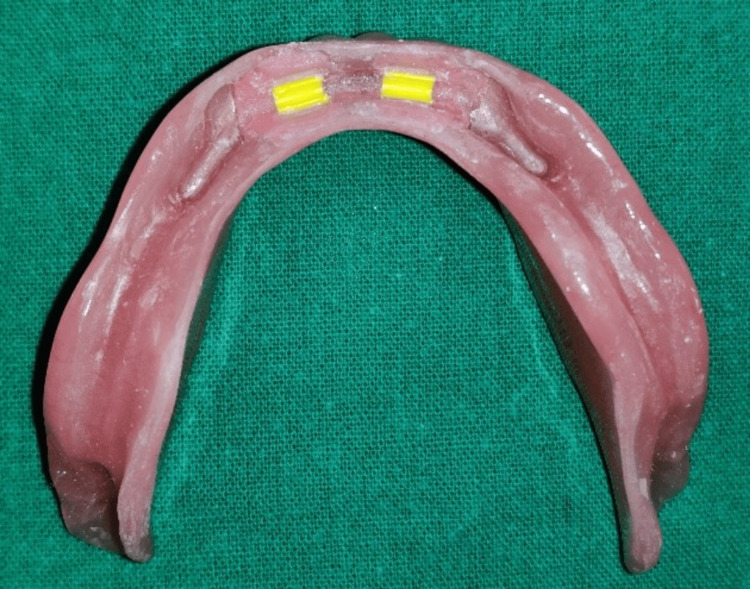
Intaglio surface of the lower denture showing incorporated and adjusted Hader bar clips

**Figure 15:**
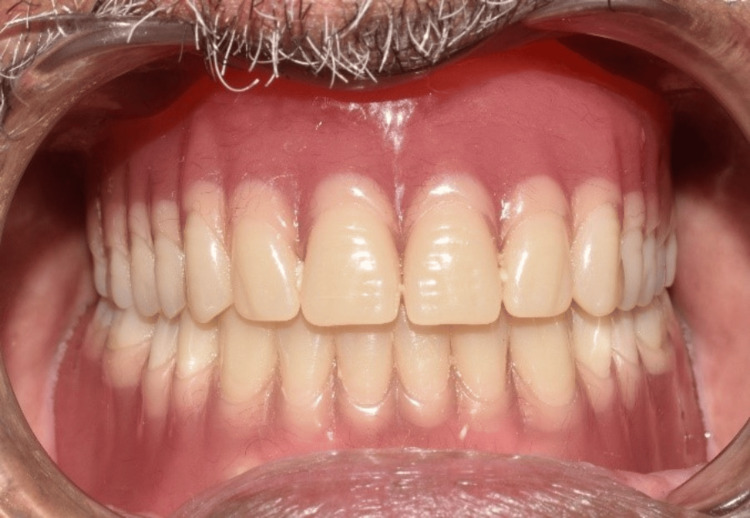
Post-maxillary and mandibular denture insertion

**Figure 16:**
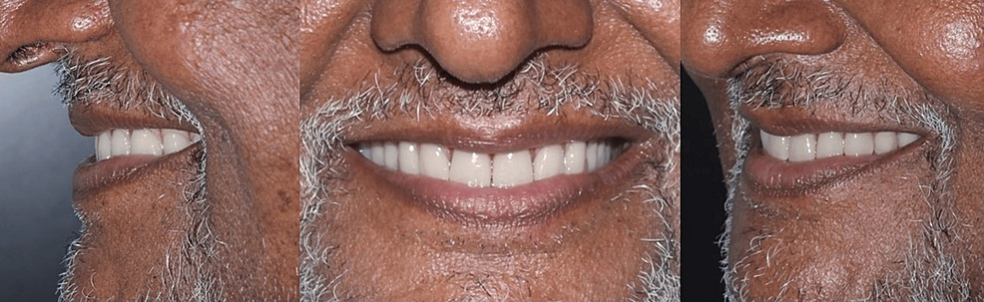
Post-denture insertion smile

A low-profile Hader bar was chosen to be fabricated and cemented over the implants. Jaw relation (Figure 9) and try-in procedures were undertaken prior to the cementation of the bar. A putty index of the bar was made and a dental stone was mixed and poured into the index and inverted over the cast (Figure 10). Packing and processing of the dentures were performed using conventional methods. On the day of delivery, the Hader bar was cemented with Glass ionomer luting cement (Meron, VOCO GmbH, Cuxhaven, Germany) (Figure 11). The clips were placed on the bar (Figure 12). The lower denture was adjusted to fit passively onto the bar and extra space was created for the pick-up of the clips (Figure 13). Occlusal adjustments were made. Cold cure acrylic (Dental Product of India, India) was mixed and applied on the intaglio surface of the denture and inserted into the patient’s mouth and the patient was instructed to close his mouth to facilitate proper pick-up of the clips (Figure 14). Necessary corrections were made until the denture had a snug fit onto the bar. Post-insertion instructions were given to the patient and he was recalled after 24 hours, 1 month, 6 months, and 1 year (Figures 15, 16).

 

## Discussion

An ailing implant is affected by peri-implant mucositis without active bone loss. In some cases, it presents with static radiographic bone loss with soft tissue pocketing and no bleeding [8]. With timely intervention and proper treatment, ailing implants can recover and remain osseointegrated with a fair long-term prognosis [9]. The patient had experienced failure with respect to three implants in the maxilla in the last eight years. Anatomic limitations and poor bone quality in the maxilla have been seen to lead to a higher failure rate of dental implants [10,11].

The patient gave a history of regular fractures and repair of the mandibular denture. On examination, reduced thickness of acrylic resin was noted at the housing area attributable to the decrease in inter-arch space caused by encroachment of the stud abutment. Four to five threads of both mandibular implants were exposed with a band of calculus around them and the implant angulations were not parallel.

The patient asked that a prosthesis be made to accommodate the current state of the implants since he was unwilling to have the implants removed and replace them with new ones. We made an attempt to minimize procedures and within the limitations of the condition of the ailing implants create a treatment plan that would maintain the health of the implants and restore esthetics and function. Monje et al. confirmed the role of implantoplasty in improving peri-implant health and several other studies verified its effects in terms of disease resolution with good implant survival rates [12,13]. Keeping this in mind, implantoplasty was performed which not only removed the exposed threads of the implants that acted like a nidus for plaque and calculus but also provided an opportunity to prepare the upper half of the implant body with a finish line and convert it into an abutment in itself. This eliminated the need for the placement of an extra abutment and provided the necessary bulk for acrylic in this area by restoring the inter-arch space. The divergence seen in the angulation of the implants was also corrected by means of implantoplasty. Incorrect angulations of implants exert unequal occlusal forces on them leading to poor long-term prognosis and causing faster wearing off of the attachments in the denture that result in a need for regular replacement of the elastic rings.

Previous studies have shown that rigid bars contribute to load sharing in the implants and minimize axial rotation and implant micromotion [11,14]. This was especially important in the present case keeping the remaining bone level and the peri-implant health in mind. Even though studies suggest that there is no significant difference in patient satisfaction with respect to various attachment systems, bars proved to be more retentive in comparison with the other systems [15-17].

**Figure 17:**
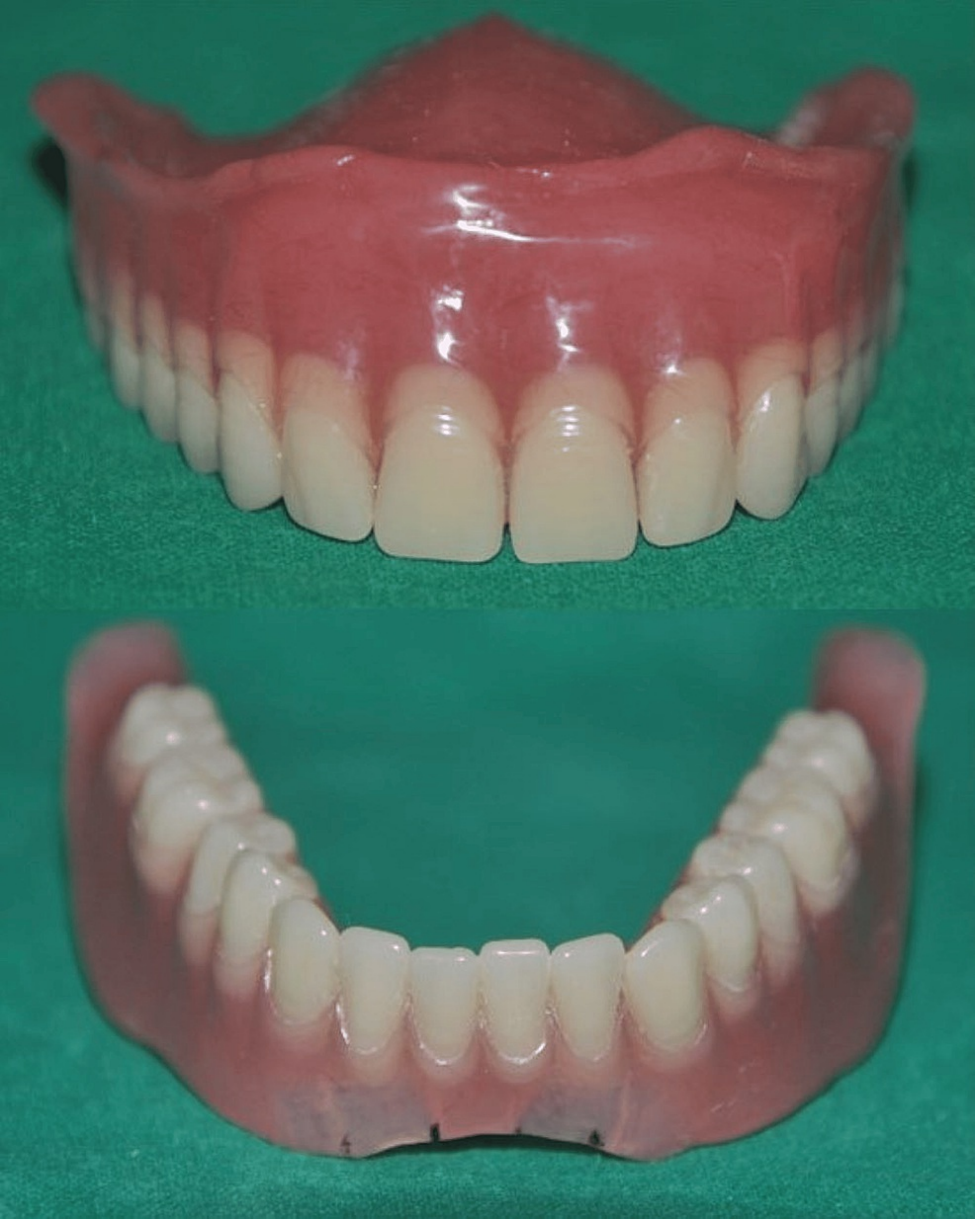
Yellow teeth set used with spacing between teeth in the maxillary denture and imbrication in the mandibular denture

**Figure 18:**
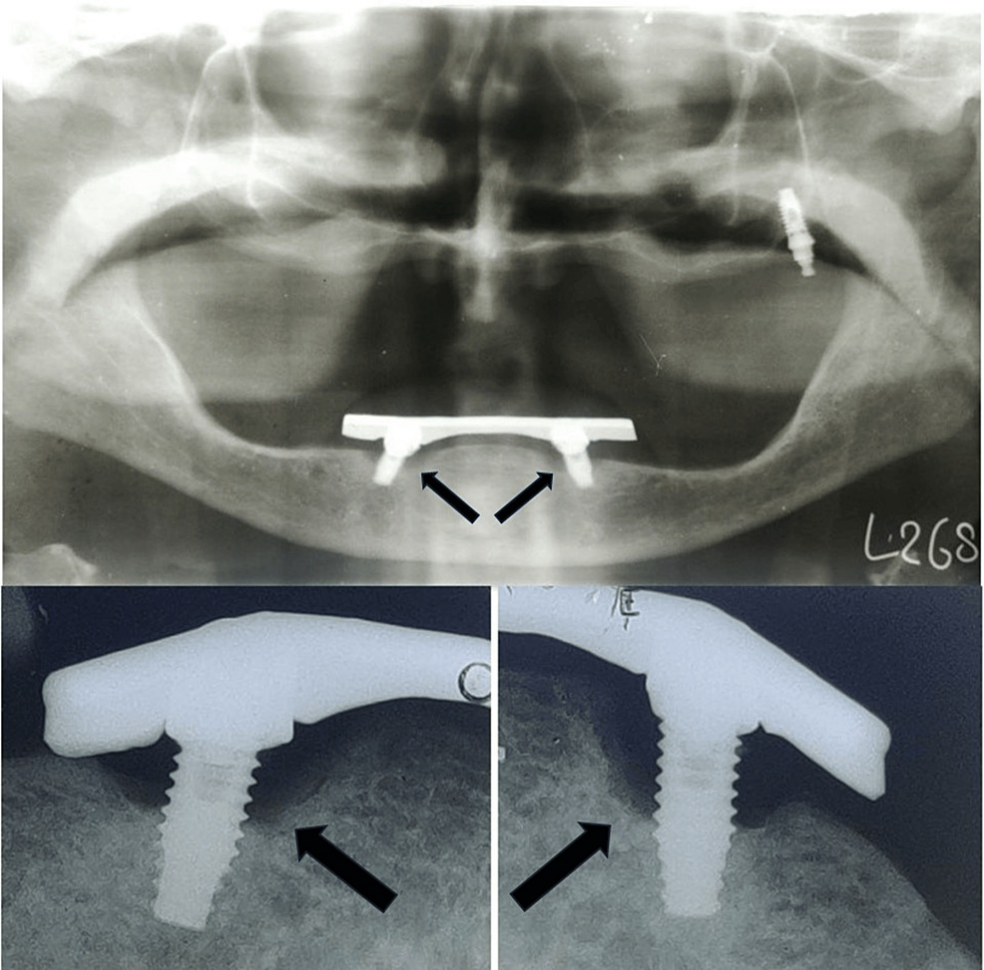
One-year follow-up radiographs showing no bone loss Arrows showing stable bone levels around mandibular implants

The incorporation of a low-profile Hader bar offered scope for the proper positioning of teeth and adequate bulk of acrylic enhancing the structural durability of the dentures as well. On the patient’s request, yellow shades of acrylic teeth with spacing in the upper denture and imbrication in the lower denture were incorporated as characterization to simulate natural teeth (Figure 17). The patient conveyed a great deal of joy and satisfaction over the new dentures' retention and esthetics. A one-year follow-up showed stable bone levels and no progression in bone loss (Figure 18).

## Conclusions

Failure is a part of clinical procedures and is not always avoidable. Keeping the needs of the patient in mind, it is crucial to provide solutions confined to their overall health and well-being. This technique showed good results in terms of patient satisfaction and outcome and can be helpful, especially in re-treatments where surgery must be avoided. The utilization of various prosthetic options in implant dentistry can help us preserve what remains and also achieve a successful functional and esthetic treatment.
